# Electricity in Medicine

**Published:** 1893-09

**Authors:** Seth M. Morris

**Affiliations:** Professor of Chemistry, Texas Medical College; Galveston


### TEXAS MEDICAL JOURNAL.

ESTABLISHED JULY, 1885.

PUBLISHED MONTHLY.—SUBSCRIPTION $2.00 A YEAR.

Vol. IX. AUSTIN, SEPTEMBER, 1893. No. 3.

### Original Contributions.

For Texas Medical Journal.

### ELECTRICITY IN MEDICINE.

BY SETH M. MORRIS, M. D., A. B.,
Professor of Chemistry, Texas Medical College.

THE ANCIENTS were aware of the fact that when amber was rubbed, it acquired the property of attracting certain light bodies, and that certain kinds of iron ore possessed the property of attracting pieces of metallic iron; and these two facts embraced all that was known, for about twenty centuries, of those two now so important branches of physical science—electricity and magnetism.

Gilbert, towards the end of the seventh century, showed that this property of amber was shared in by other substances, such as glass, sulphur and wax, and since that discovery the development of the subject has been very rapid, particularly during the present century.

Of course numerous theories of the nature of electricity have been proposed at all times, and since at first the only knowledge of electricity was confined to static phenomena, the first theories were designed to explain such phenomena, and these, the one and two fluid theories, did quite well.

That electricity is a mode of motion, there can be no doubt, and we must disabuse our minds of the notion that it is a fluid, a material something. It is a matter of daily experience with us all that mechanical motion can be converted into heat. To turn a grindstone requires the expenditure of a certain amount of energy. Press a metallic object against it, and the amount of energy required to keep up the motion is greater, and under certain conditions, a shower of sparks is given off, which shows a partial conversion of energy into heat. Imagine the condition to be different. Instead of a wheel of sandstone, we have a wheel of glass, and we press against it a pad of silk instead of a piece of metal; then instead of getting heat produced, we shall get electricity. The electricity in this case is no more a material something than was the heat produced in the former. Whenever electricity is produced, there must be an expenditure of an equivalent amount of some other form of energy elsewhere. In the frictional and induction electrical machines, it is the energy which serves to rotate the plates. In the dynamo, it is the latent energy of the coal burnt under the boiler, and in the voltaic cell, it is the energy resulting from the chemical affinity existing between the acid and the zinc. This form of energy can be developed in very many different ways—by friction, as mentioned; through induction, as in the Holtz machine and its modifications; through induction also in the dynamo; by chemical action; by the action of heat at the junction of certain metals soldered together—and according to the method adopted, we have to deal with the different kinds of electricity,—static or frictional, faradic, and galvanic.

It is not believed that there is any especial difference between these different kinds of electricity, but as their effects upon matter are very varied, more in degree than in kind, we are justified in discussing them under different headings.

### STATIC ELECTRICITY.

This is often spoken of as frictional electricity, because it can be most simply produced by rubbing certain bodies together. Induction machines,—instruments in which the principle of induction is made use of to develop the electricity,—have, because of their much greater efficiency and convenience, almost entirely taken the place of the older frictional machines. This form of electricity, to be used therapeutically, requires a very elaborate and very extensive equipment of apparatus, which, if desired, can be made very showy, and perhaps this latter fact accounts more satisfactorily for the possession of such apparatus by some physicians than does the actual utility of it. The body is a conductor, and if a person is connected with such a machine the electricity immediately escapes through him into the ground, and to prevent this it is necessary to insulate the individual by placing him on a stool or table provided with ebonite or glass legs. Now, it can be very easily demonstrated that if a conducting body of any material whatever is insulated and charged from such a machine, the charge will distribute itself over the outer surface entirely, and not penetrate in the least into the interior. There is no reason whatever to think that anything different occurs when the human is so insulated and charged, instead of a metallic one, and naturally, then, we would not expect any organs or tissues which are below the surface of the body to be in any manner affected by the application of electricity in this way, and experiment seems to confirm this supposition.

If the finger be presented to the conductor of a machine, a spark will seem to pass, and a sharp, prickling sensation, very disagreeable to some persons, will be felt.

Static electricity can, then, be used as a surface irritant, but when such irritation is desired, it can usualy be better and more economically produced in other ways. Undoubtedly a very marked mental effect can be produced in many patients by the display and operation of such apparatus, and in many cases this is certainly justifiable, but on the whole I do not think that the value of the results obtained from static electricity is sufficiently great to justify the purchase of such apparatus by the physician.

### FARADISM.

If two wires are placed side by side, near each other, but in no way connected, designated A and B, the former in connection with a galvanometer in a closed circuit and the other connected with a battery of cells with the circuit open, of course the galvanometer needle remains at rest. If, now, the circuit is closed in the wire B, it will be noticed that instantly the needle of the galvanometer is deflected, but after a few oscillations it returns to rest. A current has been produced at that instant in the wire A, but was momentary in duration, although the current continued to flow in the wire B. Now open the circuit in B, and the circuit is again deflected, but this time in the opposite direction, and again after a few oscillations it returns to rest. Another current has been produced in the wire A, again momentary in duration, but in the opposite direction. These facts were discovered by Faraday, in the year 1832, and instruments involving in their operation these principles are often called, for this reason, faradic instruments.

A faradic battery in its simplest form consists of a single cell, an induction coil, and a circuit breaker. The induction coil consists of two coils of wire wound on the same spool, thoroughly insulated from one another, the one coil, of larger and shorter wire, in connection with the cell through the circuit breaker, and the other, the smaller and longer wire, in connection with the electrodes, the whole thus forming a combination exactly analagous to the arrangement of wires mentioned above. If the electrodes be connected with only the cell of the instrument, and taken in the hands, very likely no sensation whatever will be the result. Connect together in series a number of such cells, grasp the electrodes, and very soon a burning sensation will be experienced. Break, and then make the circuit, and a sharp shock will be felt, accompanied with contractions of the muscles of the hands, or even of the arms and shoulders, according to the intensity of the current. If these makes and breaks are made very rapidly, between them the muscles have not time to relax, and a tetanic contraction results. But to produce these results, a number of cells are required.

Use a single cell connected with the primary wire of the induction coil, take in the hands the electrodes connected with the secondary coil, set in operation the circuit breaker, and instantly marked muscular contractions result. If the interruptions in the primary coil are very slow, in some respects the effects resemble those of the continuous, or galvanic current. The action of the primary current on the secondary wire is very much aided by placing in the interior of the spool a bundle of soft iron wires, which by being alternately magnetized and demagnetized produces in the secondary wire induced currents possessing the same direction as those produced by the primary curreut. As the induced currents are alternating, it is hardly practicable to measure the amount of current the patient may be receiving, and therefore the quantity used is judged of usually by the effects produced. Since with a single cell and an induction coil very marked muscular contractions can usually be easily obtained, in diseased conditions where one desires simply to produce such contractions, he would naturally use the faradic current, because of its utility and cheapness. If one of the electrodes of a faradic battery consist of a wire brush, and this be passed over the skin while the battery is in action, considerable irritation is produced; and this is of considerable value in the treatment of hysterical anæsthesia. The faradic current is preferred by many obstetricians to the galvanic for the destruction of the fœtus in cases of extra-uterine gestation. In using this current, it is of course a matter of indifference which electrode is applied to the diseased part, since the current changes its direction a great many times per second.

### GALVANISM.

I wish to introduce this part of my subject by explanations of the terms volt, ohm and ampere. A galvanic circuit can in very many respects be compared to a water works system, the battery corresponding to the pump, the current of electricity to the water, and the wire to the pipes. We speak of a pump as possessing so much power, of pipes as presenting so much resistance to the flow of water, and of a given pump delivering through a given set of pipes a certain number of gallons of water per minute. In electrical parlance, the volt is the unit of electrical pumping power or of force tending to urge the current through the wire, the ohm the unit of resistance presented by the wire to the passage of the current, and the ampere the unit of the amount of current passing through the circuit. The volt (V) then is the unit of electromotive force, the ohm (R) of resistance and the ampere (A) of current. Their values are about as follows—an ordinary Daniell's cell possesses an E. of about one volt; a copper wire one mm. in diameter and 48.5 mm. long long offers a resistance of about one ohm. Through a circuit whose E. is one, and whose resistance is one, one ampere of current will flow. There is a definite relation existing between these three values, which is best expressed by Ohm's formula—$\frac{E}{R}=A$. If any two of these values are known a third can be easily deduced.

Although the general principles underlying the action of all cells are in general the same, the cells themselves differ considerably in detail from each other. If a piece of zinc and one of copper be immersed in dilute sulphuric acid, it will be noticed that the zinc is attacked by the acid, and that hydrogen gas is given off from its surface in bubbles. Attach wires to each of these metals and bring their ends together outside the liquid, and rather curious phenomena will be the result. The wire, if very small will be heated, and may be even melted or volatilized. If it is brought very near a magnetic needle free to move pointing north and south, the needle will be deflected; while if the wire be wrapped around a piece of iron or steel, the metal will be rendered magnetic; but perhaps the most curious thing of all to be noticed is that the hydrogen is no longer given off from the surface of the zinc, but is now evolved from the face of the copper. Nevertheless, the zinc continues to dissolve while the copper is unaffected. Separate the wires and the gas is again given off from the surface of the zinc as before. Bring the wires in contact, produce a deflection of the galvanometer needle by bringing the wire near it and let it remain, when it will be noticed that the needle gradually returns to its former position, showing that the current in the wire gradually lessens and finally ceases to flow. This can be due to the consumption of the zinc, the exhaustion of the acid, or to what is called polarization, and is in fact most generally to be ascribed to the latter condition. The current in passing around the circuit has of course to pass through the cell itself, through the liquid from plate to plate. The hydrogen evolved can be seen to adhere to the copper plate, and being a bad conductor of electricity prevents the free passage of the current from the liquid to the metal. Thus the current is weakened. The accumulation of hydrogen on the copper plate also, soon virtually opposes a plate of hydrogen to the zinc, instead of copper, and this tends to send the current in the opposite direction, thus further weakening the current. To keep our cell in action then till either the acid or zinc is exhausted, it is necessary to get rid of this hydrogen, and that can be done either mechanically as in the Smee cell, or chemically as is done in the majority of cells in common use. In the bichromate cell, the potassium bichromate takes up the H. as fast as it is liberated at the copper plate, itself undergoing conversion into potassium and cromium sulphates, while in the Daniell cell the cupric sulphate is the depolarizing agent, taking up the H. with the formation of sulphuric and metallic copper, the latter depositing on the copper plate. In the Leclanche cell, the negative plate is made usually of a mixture of manganese di-oxide and powdered carbon, the former being the depolarizing agent. In the Grove cell and its modifications, the depolarizidg agent is nitric acid, which is contained in a porous vessel separating it from the sulphuric acid or exciting agent. The nitric acid takes up the H. as fast as it is liberated on the platinum or carbon plate, itself being usually reduced to nitric oxide and water. The silver chloride cell consists of a small cup or cylinder, containing chloride of silver together with a solution of either ammonium or sodium chloride, and having imbedded in the former a piece of silver wire. A cork carrying a zinc wire dipping into the fluid closes the opening of the tube or cup. The H. acts upon the silver chloride producing hydrochloric acid and metallic silver, the latter depositing upon the silver wire. In all these cells it is best to amalgamate the zinc, for then under ordinary circumstances when the circuit is open and the cell not in action, the acid will not attack the zinc, and besides no local circuits will be produced leading to the undue consumption of zinc when the battery is in action. The cells mentioned above are convenient, have considerable electromotive force, but possess qualities differing considerably from each other, leading to the selection of the one or the other in particular cases.

The bichromate and Grove cells are very troublesome to take care of, requiring to be taken down and cleaned very often, the latter in fact after each use. In every such cell it is supposed that the metal which is most attacked by the liquid becomes positively electrified, and the one least attacked, negatively. If they are connected on the outside of the liquid with a wire, immediately the negative electricity flows through the liquid from the negative plate to the zinc or positive plate, thence out into the wire connected with the zinc into the external circuit, while the positive current flows through the liquid from the zinc plate to the copper plate, out through the wire connected with it into the external circuit also, where the two currents meet, this immediately discharging the zinc and copper. But immediately the action of the liquid reproduces the condition of electrification, and impulses are again sent through the circuit, constituting the constant current. So the wire attached to the zinc plate delivers the negative current, and although the zinc is the positive plate, and is therefore called the negative pole, the wire attached to the plate constitutes the positive pole for a similar reason. When the current is spoken of without specifying which, the positive is always meant. It is extremely important in using the galvanic current to measure the amount being given the patient. A magnetic needle placed over a graduated dial with a coil of insulated wire beneath it constitutes an instrument for such measurements. This ampere meter, or milli-ampere meter accurately graduated by experiment, is placed in circuit with the patient, and from the deflection of the needle, the amount of current passing is indicated. The effects of the galvanic current are very numerous, varied and important, and may be in general classified as follows: Catalytic, Cataphoric and Catatonic.

CATALYTIC EFFECTS.

The chemical effects of the current may be discussed under this heading. Immerse in a vessel of acidulated water the electrodes of a battery of cells and the water will be decomposed, the hydrogen appearing at the negative pole and the oxygen at the positive. Very many chemical substances can be decomposed by this current when strong enough, the acid portions of the compounds appearing in general at the positive pole, and the basic portions at the negative. This can be taken advantage of to remove many new tissue formations of the body. Naevi, hair in abnormal situations, moles and various other cutaneous blemishes can be removed usually by simply inserting a fine needle and passing the current for a short time. The galvanic current has been highly recommended for the removal of urethral strictures, but it seems that this treatment has not met with much favor. Perhaps the most important therapeutical use of the current depending upon this property is the treatment of uterine fibroids. For a detailed description of the method, appliances, etc., I refer the reader to the writings of Apostoli, Keith, Massey and others. In chronic metritis and endo-metritis, cauterization of the uterine mucous membrane with the negative electrode has been highly recommended. This current has also been used with good results in cervical stenosis. Aneurism has been often treated with the galvanic current, a needle attached to the negative electrode being recommended by some, to the positive by others, for insertion into the aneurismal sac.

When a current of electricity is passed through a wire, a certain resistance is encountered and heat is produced, the amount of heat being always proportional to the resistance of the wire and the amount of the current. If the wire be long, the heat being distributed throughout the entire length, the temperature of any small section is not very high. Imagine the wire on the contrary to be short and made of such a material that the resistance is equal to that of the longer wire, and pass through it the same amount of current as before; the amount of heat being now distributed through only a short length of wire, of course each segment is highly heated. Such is the explanation of the electric cautery consisting in its simplest form of a battery, wires, and knives differing in shape, of course, according to the nature of the purpose for which they are to be used. These knives are usually made of platinum because of its very high resistance in proportion to its length and its high melting point. Carbon also possesses a very high resistance, and currents passed through it also raise its temperature, causing it to become incandescent, when it serves as a source of light. If the carbon is very small, when heated it will combine with the oxygen of the air and be consumed immediately. To prevent this, in the incandescent lamp, the carbon filament is enclosed in a glass globe from which the air is exhausted. Very small incandescent lamps are used now in connection with many surgical instruments—with the head mirror by specialists to facilitate the examination of the eye, the respiratory passages, for introduction into the æsophagus, the urethra, and the bladder as in the cystoscope, etc.

### CATATONIC EFFECTS.

The passage of a galvanic current through an organ, nerve or muscle results in a change of state of these parts, generally evident by an increase in the functional activity. If a couple of wires connected with a very delicate galvanometer be placed a short distance apart on a living nerve it can be usually demonstrated that one part of that nerve is electropositive to the other; for a current will flow through the galvanometer circuit, indicated by the deflection of the needle. If now a galvanic current is passed through another part of the same nerve, and is passed in the same direction as the normal nerve current, then this latter will be increased, while if it be passed in the opposite direction the nerve current will be decreased in intensity. It can be shown that none of the battery current escapes down the nerve affecting the instrument. This change in the electrical conditions of the nerve is known as the electrotonic state. At the same time the excitability of the nerve is considerably altered towards stimuli. If a stimulus be applied to the nerve near the cathode or negative pole of the battery, the nerve will be found more sensitive than it is when no current is passing; while on the contrary, if the stimulus is applied near the positive pole or anode, the excitability of the nerve is diminished. This condition of increased excitability near the cathode is called the electrotonic condition, and that of decreased excitability near the anode is known as the analectrotonic. These changes in the excitability of the nerves only occur when weak or moderately strong currents are employed. With very strong currents, areas of diminished excitability are present around both the cathode and the anode.

Move the muscles of the thumb voluntarily. The state of the nerve supplying these muscles has been changed; impulses have passed through them, there is more blood in them and the processes of nutrition are more active. The application of electricity to these same nerves will ordinarily produce the same effects, —viz: a contraction of the muscles supplied by them, accompanied by increased metabolism. The various organs require for their proper performance proper nutrition. Usually an increase in the amount of blood to a given part is followed by increased or improved nutrition of thát part. The galvanic current exercises this effect upon the body. Apply a moderately strong current to the skin and areas of redness will soon be noticed, especially about the negative pole, which, with strong currents may proceed to vesication. All the capillaries are dilated with blood. In a short time aftei the application, all evidences will have perhaps vanished, but some effect still remains, for if that individual four to six hours afterwards will take a warm bath, those portions of the skin, where the current was applied, will become red considerably sooner than other portions of the body.

So the galvanic current can be used in cases of paralysis where we wish not only to exercise the muscles, but also to promote their nutrition, and in many conditions where we desire to increase the metabolism of certain parts. The electrotonic effects can be taken advantage of when we desire to increase or decrease the excitability of a given nerve or organ, the one pole producing a sedative effect and the other an exciting effect. Experiments show that the galvanic current is of value in cases of post-diphtheritic paralysis. If two equally paralyzed parts are observed, the one treated with galvanism and the other not treated, the former will show evidence of improvement considerably sooner than the latter. If the one part is treated with galvanism and the other with faradism, the former will improve the more rapidly. This current is also valuable in the treatment of cutaneous anæsthesia. Suppose the continuity of the ulnar nerve to be in some way interrupted; then of course that portion of the skin supplied by it will be anæsthetic. The axis cylinders of the ulnar nerve and median form a fine net work or plexus with each other under the skin. Little filaments from the ulnar nerve pass over and communicate with the median filaments and vice versa. If these latter filaments can be set in action sensation will be conveyed by the median nerve and the area of the anæsthesia will be reduced. The frequent application of the galvanic current will often bring this about. The galvanic current is also useful in promoting nutrition in bad cases of neurasthenia. This current is very valuable in diseases of the nervous system, perhaps more for diagnosis and prognosis than for treatment. To make a complete electrical examination of a nerve or muscle it is necessary to use both the faradic and galvanic currents. The application of either pole of a faradic battery to a normal muscle or nerve will, of course, provoke a contraction. Use the galvanic current but a weak one, your apparatus being provided with a commutator, an arrangement to change the direction of the current, so that the anode can instantly be made the cathode and vice versa, thus obviating the necessity of often changing the position of the electrodes. Place the cathode on the muscle or nerve and close and open the circuit. No effect will be noticed. Gradually increase the current and soon on closure of the circuit a contraction will be produced, which is known as the cathode closure contraction—C. C. C. Opening the circuit produces no effect, and reversing and opening and closing again produces no effect. Increase the current and the C. C. C. increases, and if the current be strong enough, on reversing and closing, a contraction will result, known as the anode closure contraction—A. C. C. Sometimes contractions on the opening of the anode occur, the closing contraction at times being the more powerful, sometimes the other, but in all cases the C. C. C. is stronger than either. It requires a very strong current to provoke contractions on the opening of the cathode.

A lesion anywhere in the peripheral motor tract will be followed, of course, by degeneration of the nerve fibre and then by degeneration of the paralyzed muscles. If the lesion is above the trophic cells in the cord such a degeneration does not occur. These degenerated nerves and muscles behave very differently toward both faradism and galvanism from what they do in the normal state. The nerve itself rapidly decreases in excitability toward both the galvanic and faradic currents, and in a few weeks is entirely insensitive. Neither current applied to the nerves will provoke a contraction of the muscles. At the same time the excitability of the muscle to faradism has decreased very rapidly and finally disappears, but galvanism still produces a response, the character of which response is quite different to that which is produced when the muscle is in a normal state. In the first place, very weak currents will provoke contractions, and although as normally the C. C. C.'s are strong, the anode closure contractions are also strong, and may even exceed the former. A strong contraction is also produced on the opening of the cathode. The degenerated muscle reacts to the current very slowly and sluggishly, and unlike the normal muscle, does not usually relax immediately after the application of the current, but often continues in contraction while the current is flowing. The presence or absence of this reaction of degeneration (R. D.) locates in a general way the position of the lesion—whether above or below the trophic cells of the cords, and enables one to speak with some certainty concerning the prognosis of the disease. If the paralysis is to be long continued or is incurable, the contractions day after day to the galvanic current will become feebler, stronger currents being required to provoke them, until finally no currents whatever will be able to produce contractions. If the case is to improve, soon the contractions will partake more and more of their normal character. The C. C. C. more and more predominates, the A. C. C. becomes less in intensity and finally disappears, of course with moderate currents. The faradic excitability returns, the contractions are more vigorous and quicker, and finally the muscle returns to its normal condition. A partial reaction of degeneration in which the changes ot the muscles to galvanism are present, but in which the excitability of the nerve to galvanism and faradism are only somewhat diminished, indicates that although marked degenerative changes in the muscles have occurred, still the nerves are not much affected, the prognosis, of course, being then more favorable.

CATAPHORIC EFFECTS.

This is a property of the galvanic current in virtue of which substances in solution or suspended in liquids are carried along in the direction of the positive stream. To demonstrate this, fill a long glass tube with water slightly acidulated, place in it a drop of mercury and in the two open ends of the tube the terminals of a battery of several Grove cells, when it will be noticed that the globule of mercury moves from one end of the tube to the other in the direction of the positive current. Reverse the current and it will move back. This effect has been taken advantage of for therapeutical purposes. It is claimed that if a sponge attached to the positive pole be moistened with a strong solution of cocaine and placed over the spot to be anæsthetized, the other pole over an indiflerent portion of the body, and a moderately strong current be passed. the drug will be carried through the skin and the part rendered anæsthetic. This method has been improved upon as follows: Take a glass tube one inch in length and half an inch or more in diameter, and tie over it a piece of parchment paper. Fill the tube with a solution of the drug to be used and fit to the other end a cork with the positive wire passing through it and dipping into the liquid. Simply wet the parchment, apply it to the skin and turn on the current. Aconitia can be administered in this manner in cases of trigeminal neuralgia. Further developments in this direction are awaited.

Let us now turn our attention to a few of the problems commonly presented to a physician when selecting a battery or adapting one to a given purpose. These problems are not always very easy to solve. The kind of cell, the number as well as the manner in which they should be connected, vary with the nature of the work desired to be done. Cells can be connected together to form batteries in two general ways—in series, when the zinc of one cell is connected with the copper or carbon of the second, the zinc of this connected with the copper or carbon of the third, etc., this leaving unconnected zinc and copper wires at the ends for terminals; and in multiple arc, where all the zincs are connected together, forming one terminal, and all the coppers or zinc together forming the other. When a current passes around a circuit it, of course, passes through the wires connecting the plates outside of the liquid, and it also passes through the liquid from plate to plate. The resistance it encounters external to the cell is known as the external resistance (E. R.) of the circuit, and that which it meets inside the cell itself is called the internal resistance (I. R). The amount of current passing around any circuit is determined according to Ohm's law, by dividing the E. by the sum of the resistances of the whole circuit. It is demonstrated that connecting the cells in series increases the E. of the combination by as many times as there are cells, but at the same time increases the internal resistance by as many times as there are cells. But if the cells are connected in multiple arc the E. remains the same, while the internal resistance is diminished by as many times as there are cells. Imagine that we have five cells each having an E. of two volts, and an internal resistance of two ohms, and we wish to get the greatest amount of current through a circuit, whose external resistance is one ohm, from it as possible. Connect them in series, then the E. is five times two, or ten, which divided by eleven or the sum of the internal and external resistance, gives us five-elevenths of an ampere. Connect them in multiple, and the E. is now just two, that of a single cell, while the internal resistance is only one-fifth of two, which added to the external resistance and divided into the E. gives us ten-sevenths of an ampere, which is a greater amount of current than that obtained in the former case.

It can be equally well shown that if the E. R. be great, connecting the cells in series will give the greatest amount of current. So it can be said in general that when from a given set of cells the greatest amount of current possible is desired, if the E. R. is great, connect them in series, while if the E. R. is small, connect them in multiple. The former condition obtains usually when the current is to be applied to the human body, while the latter in the galvano cautery, the knife rarely possessing more than a fraction of an ohm of resistance. The same facts are expressed in the statement, that the best effect is obtained from a battery when the E. R. is equal to the I. R. This condition, of course, can only be usually approximated. So if the E. R. is great, connecting the cells in series increases the internal resistance; while if the E. R. is small, connecting them in multiple decreases the I. R., the two thus in both cases approximating each other. For cautery purposes, or lighting, large plates of copper and zinc are needed, the increased surface lessening the internal resistance. Storage cells or accumulators are very convenient for such purposes, and will probably come into more general use in the future. For general purposes, excluding the heating and lighting effects of the galvanic current, perhaps the Leclanche and its modifications and the silver chloride cell are most suitable, not only because of their considerable E., but because of their cheapness, portability, size and convenience.

The faradic and static currents can be said in general to possess neither catalytic nor cataphoric effects, but two physicians of my acquaintance it seems were ignorant of this fact, for they once approached me for a reason why they had obtained no good effects from the electrolytic treatment of an uterine fibroid. On inquiry, I ascertained that they had been endeavoring to resolve said tumor with an ordinary faradic battery. So a thorough knowledge of the principles of electricity is essential to a successful treatment of disease by that agent.

